# Developing Efficient Thin Film Temperature Sensors Utilizing Layered Carbon Nanotube Films

**DOI:** 10.3390/s18103182

**Published:** 2018-09-20

**Authors:** Shrutidhara Sarma, Jang Ho Lee

**Affiliations:** 1 Department of Mechanical Engineering, National Institute of Technology Delhi, Narela, Delhi 110040, India; shrutidhara.123@gmail.com; 2 School of Mechanical Engineering, Kunsan National University, Daehak-ro 588, Gunsan, Jeonbuk 54150, Korea

**Keywords:** resistance temperature detector, thin film sensors, carbon nanotube, chemical vapor deposition

## Abstract

In this paper, we present the fabrication of an efficient thin film temperature sensor utilizing chemical vapor deposited carbon nanotube (CNT) film as the sensing element on Si substrates, with diamond-like carbon (DLC):Ni as a catalyst in assisting CNT growth. The fabricated sensor showed good electrical response with change in temperature. Relative linear change in resistance of 18.4% for an increase in temperature from 22 °C to 200 °C was achieved. Various characterizing techniques, such as scanning electron microscopy (SEM) and Raman spectroscopy, were used to characterize the films. In an effort to study device performance, van der Pauw and Hall measurements were carried out to study the dependence of resistance on temperature and magnetic fields. Temperature coefficient of resistance of the sensor was calculated as 1.03 × 10^−3^/°C. All implications arising from the study are presented. The results establish the aptness of the as-grown CNT film to be used as an active sensing material in thin film temperature sensors.

## 1. Introduction

Out of all available temperature sensors, resistance temperature detectors (RTD) are the most used type as they are easy to read. Thin film sensors (TFS) are a special type of RTDs that have fast response time ranging in ~milliseconds due to the extremely small thickness of the film. Generally, TFS comprises thin metallic films (e.g., platinum, nickel, silver, gold, copper, etc.) topped over the surface of insulating, ceramic substrates (e.g., pyrex, macor, quartz, etc.). However, these sensors can be made from any conductive material. Previous research has shown the possibility of commercially available carbon nanotubes (CNT) mixed with silver pastes to be used as an active sensing element in TFS [1]. A wide range of techniques are available for thin film deposition on the substrate viz. physical/chemical vapor deposition (PVD/CVD), sputtering, spin coating, hand painting, etching etc. In order to qualify as a TFS, a sensor/gauge must obey the “semi-infinite gauge” theory. The theory states that the ratio of film thickness to substrate thickness should be such that, during the experimental run time, the heat does not have sufficient time to penetrate throughout the substrate [1]. TFS are used in a wide range of applications, including mechanical, aerospace, medical, chemical, and many other industries [1,2,3,4,5]. These sensors are very apt for such measurements because of their sufficiently short response time that is made possible by the small thickness of the films and because they bear a linear relationship between film resistance and temperature [6]. Hence, a change in temperature is sensed through a change in the film resistance, which can later be used to calculate back the amount of heat experienced by the gauge. A better thermal/electrical conductivity of the film ensures a better prediction of heat flux, especially in intricate cases where the amount of heat experienced is significantly less.

CNT has been extensively used in a number of sensors due to their exceptional transport properties (thermal/electrical), which make them excellent candidates for various applications, including temperature sensing [7,8,9]. CNTs can be used as fillers in composites to sense temperature, offering small-scale sensors with high sensitivity, fast response time, and low power consumption. In previous studies, researchers have confirmed the enhancement of thermal and electrical conduction of a silver thin film sensor by adding small amounts of commercially available multiwalled carbon nanotubes (MWCNTs) to silver [1]. The composite TFS was prepared by depositing a nanocomposite film (silver mixed with CNT) over a pyrex substrate. The nanocomposite TFS performed better than its silver counterpart when tested under similar heating environments. That work motivated us to take a step forward and synthesize CNTs in-house in order to have better control over its growth and structure. In this paper, we report the fabrication of an efficient thin film temperature sensor from layered multiwalled carbon nanotubes (MWCNTs) on DLC:Ni/Si substrates followed by their characterization. However, Ni is used this time instead of Ag because it offers the dual purpose of catalyzing CNT growth and being a good conductor.

**Figure 1:**
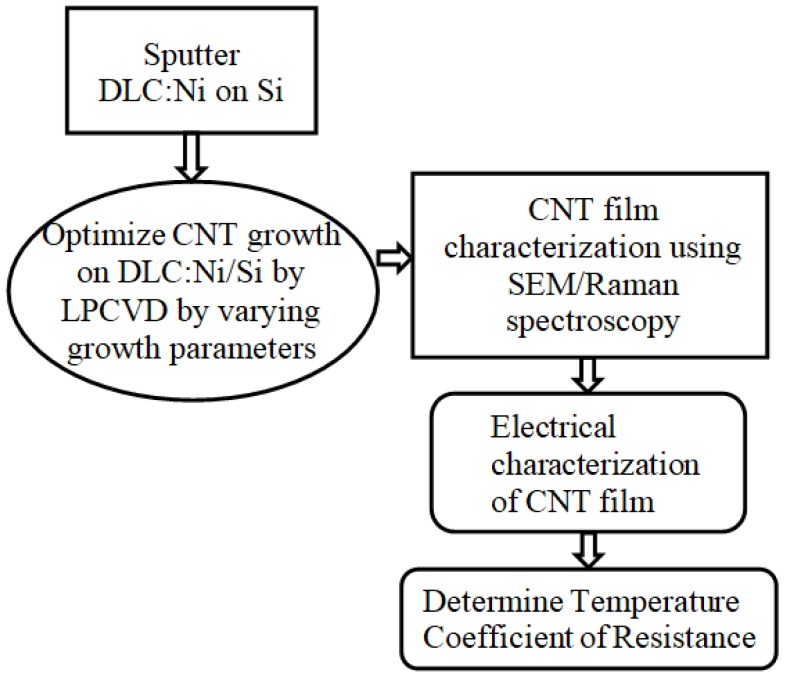
Methodology adopted for present work.

Catalytic growth using CVD process has been quoted as the easiest and most versatile of all techniques used for in situ CNT growth [10,11,12]. The catalyst layer plays a vital role in the growth and quality of the nanotubes formed. Out of several catalysts that assist in the growth of CNT, Ni is the most effective and the most easily available [10,13]. Growth of nanocomposite films consisting of metal nanospheres embedded into a dielectric matrix, such as diamond-like carbon (DLC), has been proven successful in growing high quality CNTs [10]. The present study was motivated by the question of whether CNTs are better as fillers or whether they are capable of creating independent TFS for transient heat measurements. In this study, a combination of CVD and sputtering was adopted to construct a CNT film using DLC:Ni/Si as the substrate. The quality and method of CNT formation was studied, and the ability of the CNT film to serve as the sensing element in TFS was tested. The CNT film had a temperature coefficient of resistance (TCR) value of 1.03 × 10^−3^/°C, which clearly qualifies it for being used for temperature TFS. The novelty of the work lies in fabricating a TFS using a layered, high-quality thin film of CNT, synthesized in-house, and in establishing the fact that layered CNT films are capable of acting as active sensing layers in temperature-based TFS. This work also lays the basic foundation for creating multilayered structure of nanocomposite-based TFS, with a view to quantify the enhancement in sensitivity with the number of layers and optimize them, including their thickness. The methodology adopted for the present study is shown in Figure 1.

## 2. Experimental Work

### 2.1. Substrate Preparation by Sputtering

All the untreated, unpolished Si substrates were cut into square pieces of 5 mm × 5 mm sizes beforehand to facilitate their loading into the CVD chamber (for synthesis) and on the van der Paw probe (for electrical measurements). The DLC:Ni/Si substrates in this study were prepared by sputtering a thin layer of DLC:Ni on Si using a Ni cathode at 5 W-*dc* and a carbon cathode at 50 W-*rf* in confocal geometry on a rotating target and in a flowing Ar environment for 30 min. The base pressure and working pressure for the deposition were around 2.1 × 10^(−7)^ mbar and 2.1 × 10^(−2)^ mbar, respectively. In this process, during the successive carbon–metal deposition, the DLC inhibited the growth of the metal, resulting in the formation of small metal nanospheres instead of metal layers.

### 2.2. CNT Film Synthesis

Fabrication of the samples was one of the major factors in this study as the results and analysis carried out later solely depend on the quality of the fabricated material. Use of catalyst-assisted CVD is possibly the most viable and economically attractive process for CNT synthesis. This process is crucial because it is versatile, i.e., it can be used for various carbon sources, handles various substrates, and also enables growth of CNTs in various forms. Commonly used gaseous carbon sources in catalyst-assisted thermal decomposition of hydrocarbons for CNT growths include methane, acetylene, and carbon monoxide. An inert gas is usually used as process gas to maintain a safe and clean environment during growth.

**Figure A1:**
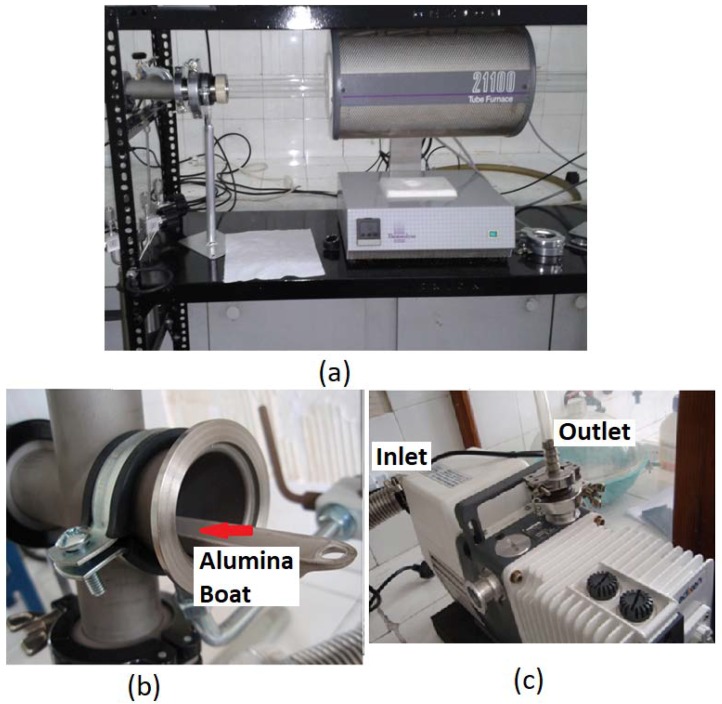
(**a**) CVD chamber used for CNT growth, (**b**) alumina boat where substrates are placed, and (**c**) vacuum pump used to evacuate the chamber.

The low pressure chemical vapor deposition (LPCVD) chamber used in the present study (refer to Appendix A, Figure A1 for picture of the setup) consisted of quartz tube coupled with a furnace, a rotary pump for evacuating, two cylinders each for the process and carbon source gases, and pressure sensors. A detector measured the temperature inside the chamber where the substrates were loaded. The gases flowed in to the chamber horizontally from the left side, and the distance between the top and bottom electrodes was around 10 cm. Two gases were used during the CNT synthesis: Ar as the precursor and acetylene as the carbon source gas. The DLC:Ni/Si substrates were placed on an alumina boat and introduced into the quartz chamber. The system was subsequently vacuumed up to a base pressure of 6.6 × 10^−3^ mbar. Once the stable pressure was reached, the chamber was filled with Ar and heated in ramp up to 700 °C, a temperature where the DLC:Ni got completely graphitized. Ar was introduced into the chamber at a flow rate of 100 sccm when the working pressure rose up to 1.7 mbar. When acetylene encounters NiC_3_ at such high temperature and low pressure, acetylene is broken apart at the surface of the catalyst (Ni) particle, and the carbon is transported to the edges of the particle where it forms the nanotubes [10]. A flow rate of 20–22 sccm of acetylene was used for a total time of 5, 15, and 40 min for CNT growth. Several trials were made in order to find the optimized combination of growth temperature, deposition rate, and growth time to achieve continuous and high quality CNT growth on the DLC:Ni layer sitting over the Si substrate. Finally, the samples were unloaded when the temperature inside the chamber came well below 80 °C.

### 2.3. Characterization

For obtaining morphological, microstructural, and analytical information, the obtained CNT films were observed using a high-resolution scanning electron microscope (SEM, JEOL-JSM6390 scanning electron microscope using a W-filament).

Raman spectral analysis of the samples was performed in the back-scattering geometry using a LabRam HR spectrometer (HORIBA) equipped with a Peltier-cooled charge-coupled detector. A 100× standard objective (~1 μm focus spot) with a laser power lower than 1 mW was used to avoid any laser-induced heating effects, with excitation provided by the 514.6 nm line of a diode-pumped solid-state laser.

**Figure A2:**
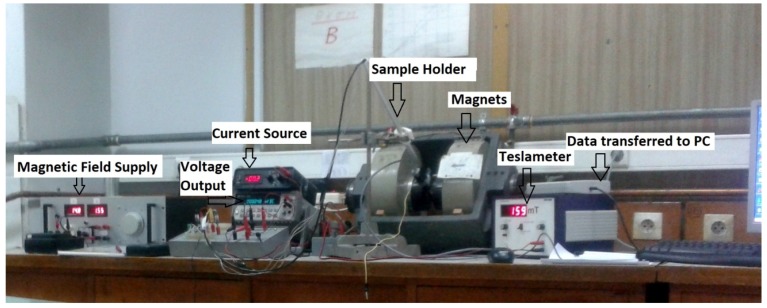
Van der Pauw and Hall coefficient measurement setup.

**Figure A3:**
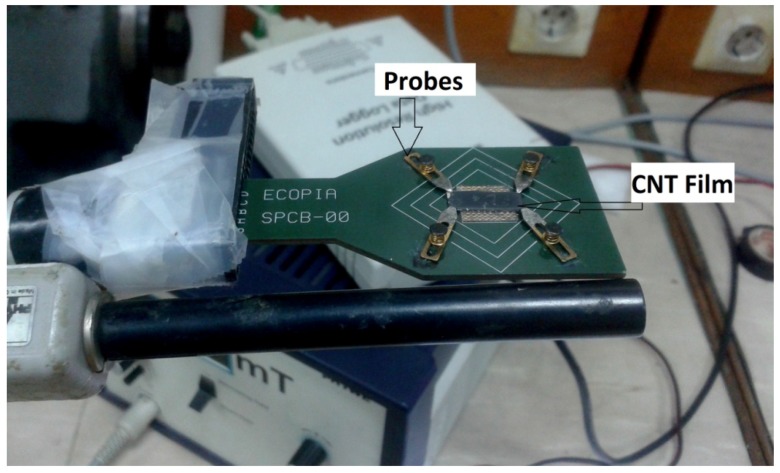
Four probes on the CNT film.

**Figure A4:**
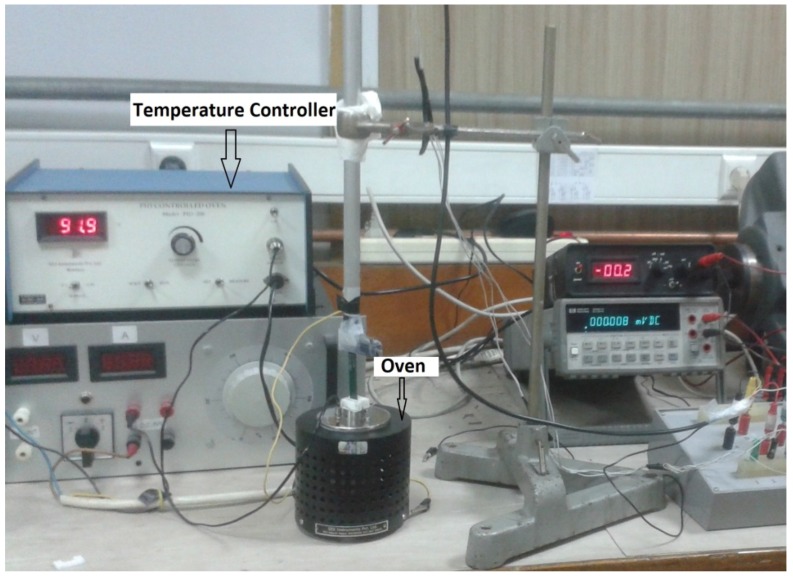
Static calibration setup for temperature coefficient of resistance (TCR) determination of CNT film.

A four-point probe setup was used to measure the electrical conductivity and mobility of the samples (refer to Appendix A, Figure A2 for picture of the setup). The four probes held the samples at the periphery with pressure instead of glue, which reduced contact resistance that would have been caused otherwise by conductive silver epoxy (refer to Appendix A, Figure A3). The setup consisted of a probe with four tungsten metal tips to hold the samples, a Teslameter along with a magnetic probe to measure the applied magnetic field, a variable current source to apply current through the samples, a multimeter (HP 34401 A), and a high resolution data logger (PICO) to transfer the data into the PC. A PID-controlled oven (PID-200) was connected to the four probe setup that allowed heating of samples up to 200 °C in order to measure their TCR. Square samples of size 5 mm × 5 mm were used for the measurements. Current was passed across two probes, the corresponding voltage across the other two probes was noted, and the sheet resistance was measured. The complete set up for van der Pauw and Hall measurements are shown in Appendix A, Figure A4.

## 3. Results and Discussion

### 3.1. CNT Morphology and Microstructures 

**Figure 2:**
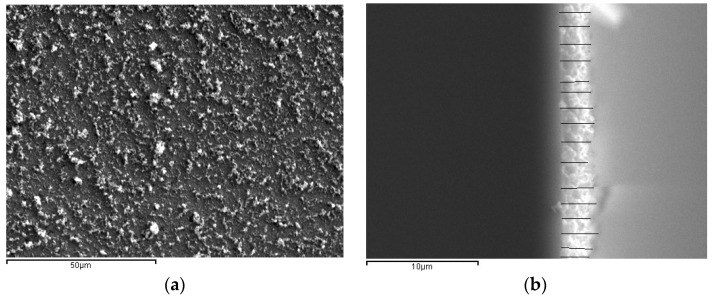
SEM images of carbon nanotube (CNT) film (**a**) plain view at 1000× and (**b**) cross-sectional view at 4000× magnification.

Uniform growth of CNTs is a challenging job as they are sensitive to many factors, including surface roughness, flow of source gas, inside temperature, etc. SEM analysis carried out on the CNT samples revealed a forest-like growth of the MWCNTs. Figure 2 shows the planar view of the CNT film obtained through SEM. The film had a thickness in the range of 2.65–2.75 µm, i.e., 2.7 µm on average. Numerous nanotubes could be viewed in the photographs, and the diameter of the individual CNTs in DLC: Ni/Si substrates were calculated as 240 nm on average. Cross-sectional view obtained through SEM helped in determining the film thickness as well as measuring the nanotube diameter.

### 3.2. Raman Spectroscopy Results

**Figure 3:**
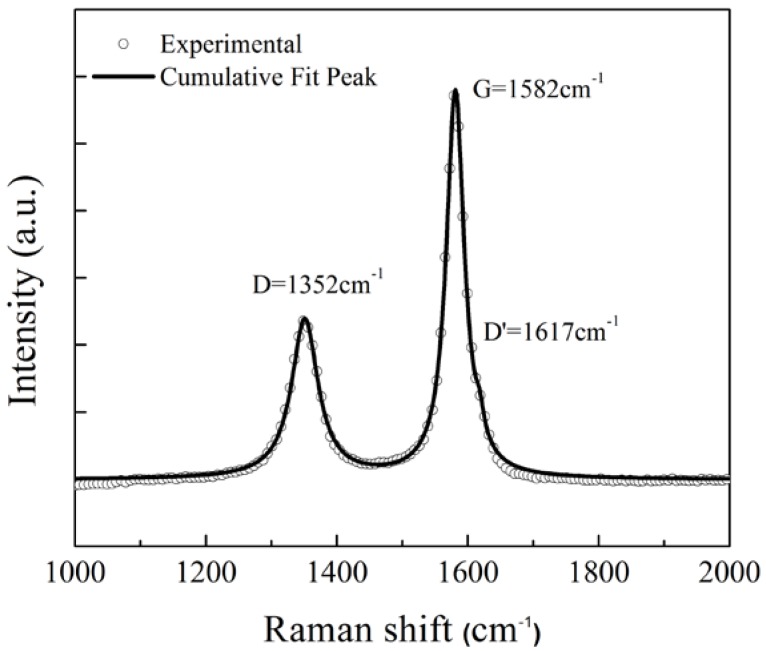
Raman spectroscopy of CNT produced by chemical vapor deposition (CVD).

Raman spectroscopy was carried out to evaluate the structural quality of the CNT produced. The Raman spectrum of CNT carried two dominating features, i.e., two sharp Raman peaks, namely G (graphite) and D (disorder) bands. The G band consisted of two peaks—G and D′ (the most prominent ones)—which were related to the lattice vibration of sp2 bonds of carbon [14]. For CNT/DLC:Ni film, the modes G and D′ occurred at 1582 cm^−1^ and 1617 cm^−1^, while the D peak was observed at 1352 cm^−1^. The ratio of intensity of D and G bands (I_D_/I_G_) revealed the degree of disorder in the graphitic sheets and the alignment in CNT [15,16]. A lower I_D_/I_G_ ratio of 0.6 and sharper D, G peaks in the CNT/DLC:Ni film confirmed aligned and ordered CNTs. The Full Width Half Maximum (FWHM), the peak position, and the I_D_/I_G_ ratios are listed in Table 1. The measured peaks were fitted with Lorentzians. Several measurements were performed on the same spot and at different spots on the same sample to confirm the film quality. Figure 3 shows the Raman spectra of the CNT/DLC:Ni film. The agreement of position and intensity of Raman peaks with those cited in the literature proved the concreteness and high quality of the CNTs produced [16,17].

### 3.3. Electrical Characterization

#### 3.3.1. Hall Coefficient Measurement through van der Pauw

**Figure 4:**
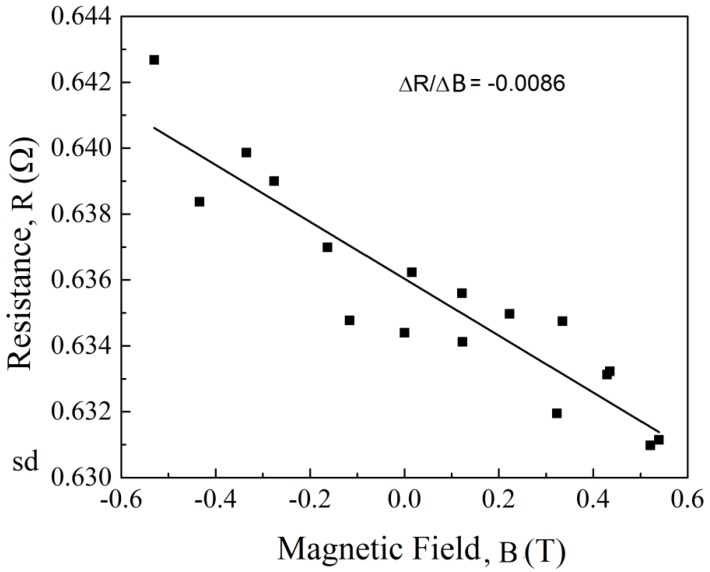
Resistance vs. magnetic field of CNT film.

The conduction mechanism of any material, especially composites, is very complicated. Many factors can influence the electrical properties, and these should be considered carefully during designing an effective conductive network. The sheet resistance and Hall coefficients of the films were measured using a van der Pauw setup with four leads attached on the four corners of 5 × 5 mm^2^ square samples. The voltage–current relationship was measured thrice for each sample, each time using two different points (where the leads touched the sample) to pass current and measure output voltage difference between the rest. The initial resistance of the CNT/DLC:Ni/Si was measured as 0.64 Ω. The presence of n-type carriers was detected through Hall measurement, and the net carrier mobility in the CNT/DLC:Ni held a value as low as −28.56 cm^2^/Vs. Figure 4 shows the change in resistance (in ohms) with respect to the change in magnetic field (in Tesla) of the samples. A fair number of sample points were collected by varying the magnetic field, and a best fit line (linear fit) was used to calculate the slope, which is an essential parameter in the calculation of carrier mobility and conductivity of the film under study. The resistances, hall coefficients, electron mobility, resistivity, conductivity, and sensitivity of the sample are listed in Table 2.

#### 3.3.2. TCR of CNT Film

**Figure 5:**
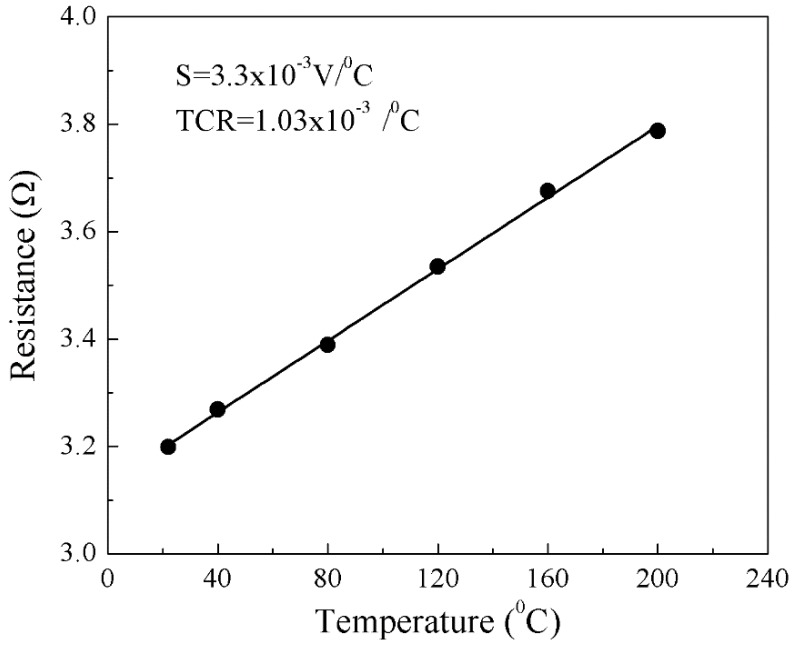
Resistance vs. temperature of CNT film.

The TCR of an RTD determines its change in resistance per unit change in temperature and is a measure of its performance. A van der Pauw setup together with an oven serves the purpose of calibrating the sensor for temperature and determines its TCR. For this, the sample holder together with the sample was introduced in the temperature-controlled oven. At a certain temperature when the environment inside the oven was stabilized, the change in resistivity with increase in input current was noted, and the procedure was repeated for every temperature step (refer to Appendix A, Figure A4). The temperature inside the oven was varied from room temperature to 200 °C. The highest temperature that could be applied was 200 °C, as limited by the instrument. Keeping the temperature constant, the applied current was changed from 0 to 120 mA (as limited by the instrument), and the change in voltage was noted. Being a passive sensor, TFS required activation by passing a small current (~2 mA) through it. The variation of resistance with the amount of input current was linear, so was the variation of resistance with increase in temperature when the current was fixed. The change of film resistance with increase in temperature is shown in Figure 5.

The TCR of the CNT film based TFS came out as 1.03 × 10^−3^. The higher sensitivity (3.3 × 10^−3^ V/°C) for the CNT/DLC:Ni/Si film was certainly due to the better quality of the CNTs grown, as confirmed by Raman spectroscopy (lower I_D_/I_G_ value). Hence, an efficient TFS was achieved through synthesizing high quality CNT film over a DLC:Ni/Si substrate.

## 4. Conclusions and Future Work

To conclude, growth of a continuous uniform CNT film was achieved successfully using DLC:Ni as a catalyst over Si substrate to be used as a TFS. The growth process was optimized by varying the growth parameters, such as deposition time, furnace temperature, and ratio of process gas to carbon source gas, in order to achieve a continuous CNT growth. A forest-like growth of CNT was observed through SEM. The sharpness of D and G peaks and the low ratio of their intensity during Raman spectroscopy confirmed the high quality of the CNTs grown. Sensitivity of the film towards temperature was proven through the van der Pauw measurement, and the type of mobility was determined using Hall coefficient measurement. The fabricated temperature sensor with CVD-grown CNT film as the sensing element was sensitive to change in temperature. The TCR of the CNT film was measured as 1.03 × 10^−3^, which was reasonable for a thin film TFS. The linear variation of resistance with temperature was another important feature of the sensor. The ability of the layered CNT film to be used as active sensing layer in thin-film-based temperature sensors was thus established through this work. The influence of the DLC:Ni catalyst layer on the sensor measurements as a control experiment is being contemplated in immediate future work. This will help in quantifying the sensor output with respect to the catalyst layer thickness.

## Figures and Tables

**Table 1:** Peak position, FWHM, and intensity of D and G bands (I_D_/I_G_) ratios for the CNT film.

Sample		D	G	D′	I_D_/I_G_
CNT/DLC:Ni/Si	Peak center	1352.38	1582.12	1617.2	0.6
	FWHM	51.32	32.81	23.56	

**Table 2:** Table displaying resistivity, conductivity, and carrier mobility of the films.

Sample	Film Thickness (m)	Resistivity (ρ) in Ω.cm	Sensitivity in V/˚C	Conductivity (σ) in Ω.cm^−1^	Carrier Mobility (μ) in cm^2^/Vs
CNT/DLC:Ni/Si	2.70 × 10^−6^	8.64 × 10^−4^	3.3 × 10^−3^	1157.41	−28.5574

## References

[1] Sarma S., Unal A., Sahoo N. (2015). Thin Film Gauges using Carbon Nanotubes as Composite Layers. ASME J. Eng. Mater. Technol..

[2] Henze M., Bogdanic L., Muehlbauer K., Schnieder M. (2013). Effect of the Biot Number on Metal Temperature of Thermal Barrier Coated Turbine Parts—Real Engine Measurements. ASME J. Turbomachin..

[3] Patil P.S., Belsare S.N., Borse S.L. (2012). Analysis of Internal Combustion Engine Heat Transfer Rate to Improve Engine Efficiency, Specific Power & Combustion Performance Prediction. Int. J. Mech. Eng. Technol..

[4] Mortazavinatanzi S., Rezaniakolaei A., Rosendahl L. (2018). Printing and Folding: A Solution for High-Throughput Processing of Organic Thin-Film Thermoelectric Devices. Sensors.

[5] Jiang H., Huang M., Yu Y., Tian X., Zhao X., Zhang W., Zhang J., Huang Y., Yu K. (2018). Integrated Temperature and Hydrogen Sensors with MEMS Technology. Sensors.

[6] Sahoo N., Saravanan S., Jagadeesh G., Reddy K.P.J. (2006). Simultaneous Measurement of Aerodynamic and Heat Transfer Data for Large Angle Blunt Cones in Hypersonic Shock Tunnel. Sadhana.

[7] Bartolomeo A.D., Sarno M., Giubileo F., Altavilla C., Iemmo L., Piano S., Bobba F., Longobardi M., Scarfato A., Sannino D. (2009). Multiwalled Carbon Nanotube Films as Small Sized Temperature Sensors. J. Appl. Phys..

[8] De Volder M., Reynaerts D., Hoof C.V., Tawfick S., Hart A.J. (2010). A Temperature Sensor from a Self-Assembled Carbon Nanotube Microbridge. IEEE Sens..

[9] Sibinski M., Jakubowska M., Sloma M. (2010). Flexible Temperature Sensors on Fibers. Sensors.

[10] Panagiotopoulos N.T., Diamanti E.K., Koutsokeras L.E., Baikousi M., Kordatos E., Matikas T.E., Gournis D., Patsalas P. (2012). Nanocomposite Catalysts Producing Durable, Super-Black Carbon Nanotube Systems: Applications in Solar Thermal Harvesting. ACS Nano.

[11] Kong J., Cassell A.M., Dai H. (1998). Chemical Vapor Deposition of Methane for Single-Walled Carbon Nanotubes. Chem. Phys. Lett..

[12] Kong J., Hyongsok T.S., Cassell A.M., Quate C.F., Dai H. (1998). Synthesis of Individual Single-Walled Carbon Nanotubes on Patterned Silicon Wafers. Nature.

[13] Ducati C., Alexandrou I., Chhowalla M., Amaratunga G.A.J., Robertson J. (2002). Temperature Selective Growth of Carbon Nanotubes by Chemical Vapor Deposition. J. Appl. Phys..

[14] Dresselhaus M.S., Jorio A., Saito R. (2010). Characterizing Graphene, Graphite, and Carbon Nanotubes by Raman Spectroscopy. Ann. Rev. Cond. Matter Phys..

[15] Ferrari A.C., Robertson J. (2000). Interpretation of Raman Spectra of Disordered and Amorphous Carbon. Phys. Rev. B.

[16] Chhowalla M., Teo K.B.K., Ducati C., Rupesinghe N.L., Amaratunga G.A.J., Ferrari A.C., Roy D., Robertson J., Milne W.I. (2001). Growth Process Conditions of Vertically Aligned Carbon Nanotubes using Plasma Enhanced Chemical Vapor Deposition. J. Appl. Phys..

[17] Bokobza L., Zhang J. (2012). Raman Spectroscopic Characterization of Multiwall Carbon Nanotubes and of Composites. Express Polym. Lett..

